# Call Me, Maybe? A Multidisciplinary Approach to Identifying and Reporting MIS-C

**DOI:** 10.1017/ash.2025.380

**Published:** 2025-09-24

**Authors:** Kristina Herndon, Lea Kendrick

**Affiliations:** 1 Children’s Healthcare of Atlanta

## Abstract

**Background:** Sars-CoV-2, better known as COVID-19, emerged in late 2019 and was declared a pandemic in March 2020. In April 2020, it was noted that multiple children were having serious inflammatory symptoms after being diagnosed with or exposed to COVID-19. The Centers for Disease Control and Prevention (CDC) defined MIS-C with a specific case definition in May 2020 that included fever, clinical severity resulting in hospitalization, evidence of systemic inflammation, involvement of at least two organ system, and no alternative plausible diagnosis. A revised case definition was issued in January of 2023 which included subjective or documented fever, clinical severity requiring hospitalization or death, elevated C-reactive protein, new onset manifestations of 2 or more organ systems, and absence of a more likely alternative diagnosis. 2023 case definition eliminates involvement of renal, respiratory or neurologic systems. Some cases reported under the original case definition would not fit the 2023 case definition. Multisystem Inflammatory Syndrome in Children (MIS-C) symptoms can include, but are not limited to, fever, red eyes, abdominal pain, nausea, vomiting, diarrhea, and organ dysfunction. Cases have varied from mild to severe. The first case of MIS-C seen at Children’s was admitted in April of 2020. **Method:** Diagnosing MIS-C is not always clear cut. To help with properly diagnosing cases, a multi-disciplinary team (Team) was formed to review cases regularly before reporting to the state and CDC. The team included Infection Prevention Epidemiologists, Pediatric Infectious Disease Physicians, General Pediatric Physicians, Pediatric Cardiologists, members of the Georgia Department of Public Health State Epidemiology staff, and members of the CDC staff. **Results:** Initially, the Team held weekly meetings to discuss cases. As cases declined in frequency, meetings were switched to bi-weekly, then monthly, and are now held ad hoc. Between April 2020 and December 2023, a total of 1339 potential cases were reviewed, and 485 cases were confirmed as meeting case definition. Of the confirmed cases, 216 required admission to an intensive care unit (ICU). **Conclusion:** According to CDC representatives, the number of cases reported from Children’s was significantly higher than from comparable pediatric facilities in other parts of the country. We believe this is due to the collaborative nature of our review process and our diligence in ensuring that all patients that could possibly meet definition were reviewed, regardless of clinical diagnosis. All patients were discussed to ensure a consensus was reached prior to reporting.

